# Assessment of serum vitamin D levels in children aged 0–17 years old in a Chinese population: a comprehensive study

**DOI:** 10.1038/s41598-024-62305-7

**Published:** 2024-05-31

**Authors:** Yuan Zhang, Lijun Zhou, Yaqiong Ren, Hongyan Zhang, Wenna Qiu, Hongying Wang

**Affiliations:** 1 Laboratory of Pediatric Research, Children’s Hospital of Wujiang District, Suzhou, 215234 Jiangsu People’s Republic of China; 2 Department of Clinical Laboratory, Children’s Hospital of Wujiang District, Suzhou, 215234 Jiangsu People’s Republic of China; 3 Institute of Pediatric Research, Children’s Hospital of Soochow University, Suzhou, 215025 Jiangsu People’s Republic of China

**Keywords:** Vitamin D, Deficiency, Insufficiency, Children, Nutrition, Risk factors, Epidemiology

## Abstract

Vitamin D deficiency and insufficiency pose global public health challenges, yet research on serum vitamin D levels in the 0–17-year-old age group in southeastern China remains limited. This study aimed to fill this gap by investigating serum 25(OH)D levels in children in the region aged 0–17 years, contributing crucial data for understanding vitamin D nutritional status. Liquid chromatography‒mass spectrometry/mass spectrometry (LC‒MS/MS) technology was used. Vitamin D testing was integrated into routine diagnostic procedures for 11,116 children in Wujiang District, Suzhou City. Among the 0–17-year age group, comprising 6348 boys and 4768 girls, the prevalence of serum 25(OH)D deficiency and insufficiency was 21.4% and 31.0%, respectively. The median serum 25(OH)D concentration was 29.72 ng/mL (21.84–39.84 ng/mL) in boys compared to 28.48 ng/mL (20.65–39.23 ng/mL) in girls. Seasonal variations were observed, with median serum 25(OH)D concentrations of 29.02 ng/mL (20.73–39.72 ng/mL) in spring, 28.79 ng/mL (21.53–39.37 ng/mL) in summer, 30.12 ng/mL (22.00–39.70 ng/mL) in autumn, and 28.58 ng/mL (19.97–39.46 ng/mL) in winter. Statistically significant differences were noted in the serum 25(OH)D levels during autumn and winter. In conclusion, the rate of adequate vitamin D levels in local children was 47.5%, revealing a relatively high prevalence of vitamin D deficiency (21.4%) and insufficiency (31.0%), especially during the post-preschool period. Advocating for vitamin D supplementation in children is crucial for ensuring adequate vitamin D support.

## Introduction

The current status of vitamin D insufficiency and deficiency is a widely recognized public health concern^1–3^. The prevalence of vitamin D deficiency is a common concern due to various factors, such as seasonal changes, lifestyle habits, and environmental conditions in different regions. In particular, at high latitudes, insufficient sunlight during the winter limits the opportunity for the skin to synthesize vitamin D. Modern indoor living and cultural differences further contribute to reduced natural exposure. This global challenge is particularly pronounced in its impact on children, because vitamin D deficiency can adversely affect bone health, immune system function, and overall growth and development. From the perspective of childhood growth, vitamin D deficiency may have negative implications for bone development and hardening, increasing the risk of children developing rickets^4,5^. Moreover, vitamin D deficiency is correlated with an elevated risk of autoimmune diseases, asthma, and other chronic conditions^6–9^. Insufficient vitamin D in children may lead to a weakened immune system, increasing susceptibility to infectious diseases, particularly respiratory infections^4,10,11^. Vitamin D deficiency poses a potential threat to the physical health and quality of life of children.

To address this issue, scholars from various countries worldwide have conducted surveys to assess vitamin D status. For instance, NHANES estimates from 2001 to 2010 revealed a prevalence of 28.9% for vitamin D deficiency^12^. The DEGS1 survey indicated that 61.6% of participants in Germany had serum 25(OH)D levels below 20 ng/mL^13^. According to the KNHANES, the prevalence of vitamin D deficiency among Korean males and females was reported to be 65.7% and 76.7%, respectively^14^.

According to CNNHS data, the prevalence of vitamin D deficiency is relatively elevated among Chinese adults aged 60 and above, with a rate of 34.1% for males and 44.0% for females^15^. For pregnant women, the vitamin D deficiency rate was 74.83% from 2010 to 2012 and increased to 87.43% from 2015 to 2017^16^. Among children aged 6–17 years old, the prevalence of vitamin D deficiency was 53.2%^17^. Multiple research reports suggest that approximately one billion people across various age groups worldwide are deficient or insufficient in vitamin D^18^. Nevertheless, existing studies on the vitamin D status of individuals aged 0–17 years in southeastern China are currently limited.

This study was aimed at assessing the vitamin D levels of the pediatric population (0–17 years old) in Wujiang district, Suzhou City, southeastern China. Additionally, gaining insights into how age, sex, and season impact vitamin D levels is highly valuable. This study can provide valuable insights for health care professionals to offer improved vitamin D supplementation recommendations, contributing collectively to addressing the issue of vitamin D deficiency. The ultimate goal is to ensure optimal health conditions for children across different age groups.

## Results

### Vitamin D status in different sex groups

This study included a total of 6348 boys and 4768 girls aged 0–17 years. Serum 25(OH)D concentrations were assessed, and sex-based analysis revealed that the median serum 25(OH)D concentration in boys was 29.72 ng/mL (IQR 21.84–39.84 ng/mL), whereas in girls, it was 28.48 ng/mL (IQR 20.65–39.23 ng/mL). The serum 25(OH)D concentration in boys was significantly greater than that in girls. When categorized into deficiency, insufficiency, and sufficiency groups, there was a statistically significant difference in serum 25(OH)D levels between boys and girls. The occurrence of serum 25(OH)D deficiency was greater in girls (31.7%) than in boys (30.6%), and similarly, the prevalence of serum 25(OH)D deficiency was greater in girls (23.0%) than in boys (20.2%) (Table 1).

**Table 1:** Prevalence of vitamin D concentration and levels by sex, age group and season.

		Median(IQR:ng/mL)		Vitamin D group							
				*P*^1^	Deficiency n = 2380		Insufficient n = 3454		Sufficiency n = 5282		*P*^2^
					n	%	n	%	n	%	
Gender	Boy	29.72	(21.84–39.84)		1281	20.2%	1942	30.6%	3125	49.2%	
	Girl	28.48	(20.65–39.23)	< 0.001	1099	23.0%	1512	31.7%	2157	45.2%	< 0.001
Age	< 1	37.36	(28.89–48.00)		222	5.2%	1010	23.5%	3073	71.4%	
	1–3	32.33	(24.70–42.08)		247	12.9%	562	29.3%	1106	57.8%	
	3–6	25.41	(19.27–32.59)		532	27.5%	768	39.7%	633	32.7%	
	6–12	20.92	(16.22–27.05)		1269	45.7%	1049	37.8%	460	16.6%	
	12–17	19.18	(15.31–23.57)	< 0.001	110	59.5%	65	35.1%	10	5.4%	< 0.001
Season	Spring	29.02	(20.73–39.72)		546	23.3%	705	30.1%	1092	46.6%	
	Summer	28.79	(21.53–39.37)		725	20.3%	1195	33.4%	1656	46.3%	
	Autumn	30.12	(22.00–39.70)		623	19.2%	991	30.5%	1636	50.3%	
	Winter	28.58	(19.97–39.46)	< 0.001	486	25.0%	563	28.9%	898	46.1%	< 0.001

*P*^1^: The *Mann–Whitney* U rank-sum test was utilized for sex*.* The *Kruskal–Wallis* H test was applied for age and season.

*P*^2^: *Chi-square* test.

### Vitamin D status across different age groups

**Figure 1:**
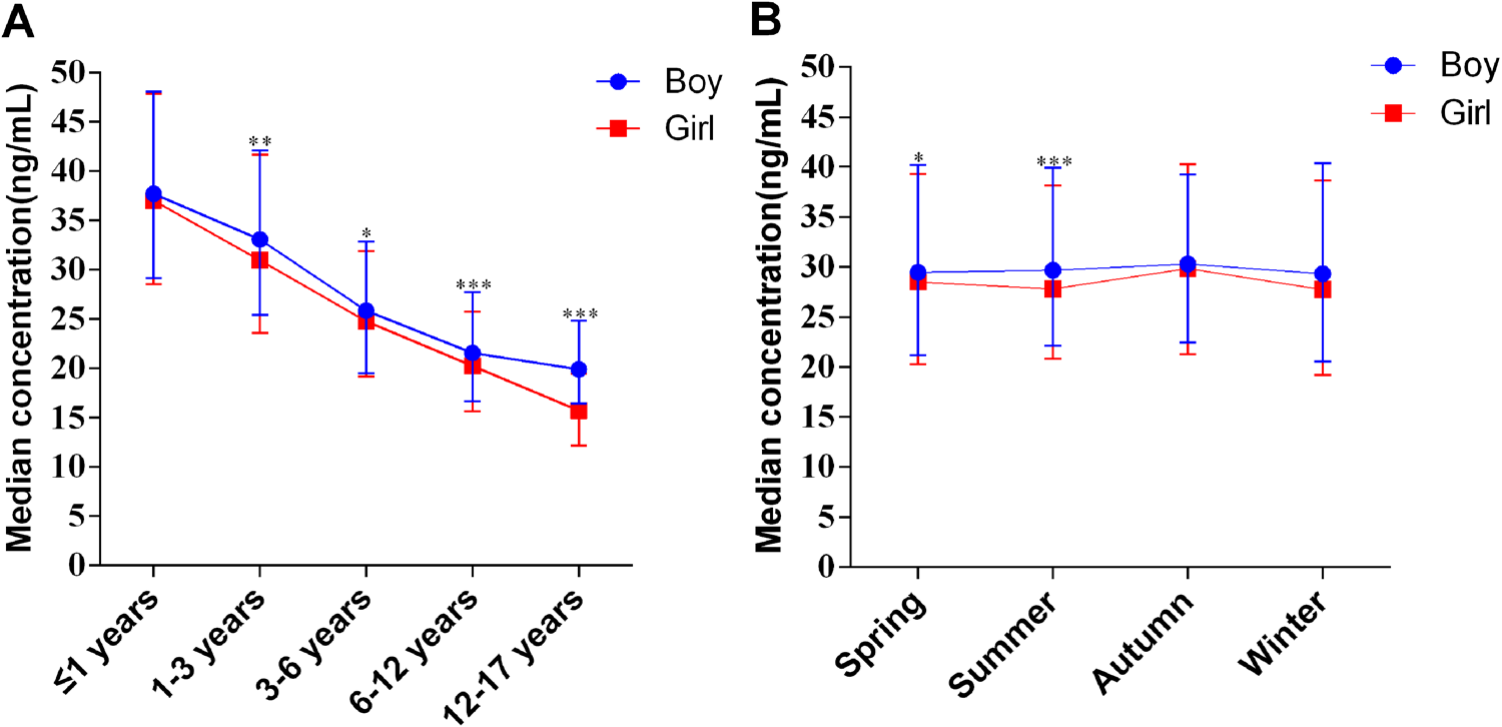
(**A**) The distribution of serum 25(OH)D concentrations stratified by age and sex. (**B**) Distribution of serum 25(OH)D concentrations stratified by season and sex. Error bars represent the interquartile range (IQR). *P < 0.05. **P < 0.01. ***P < 0.001.

**Figure 2:**
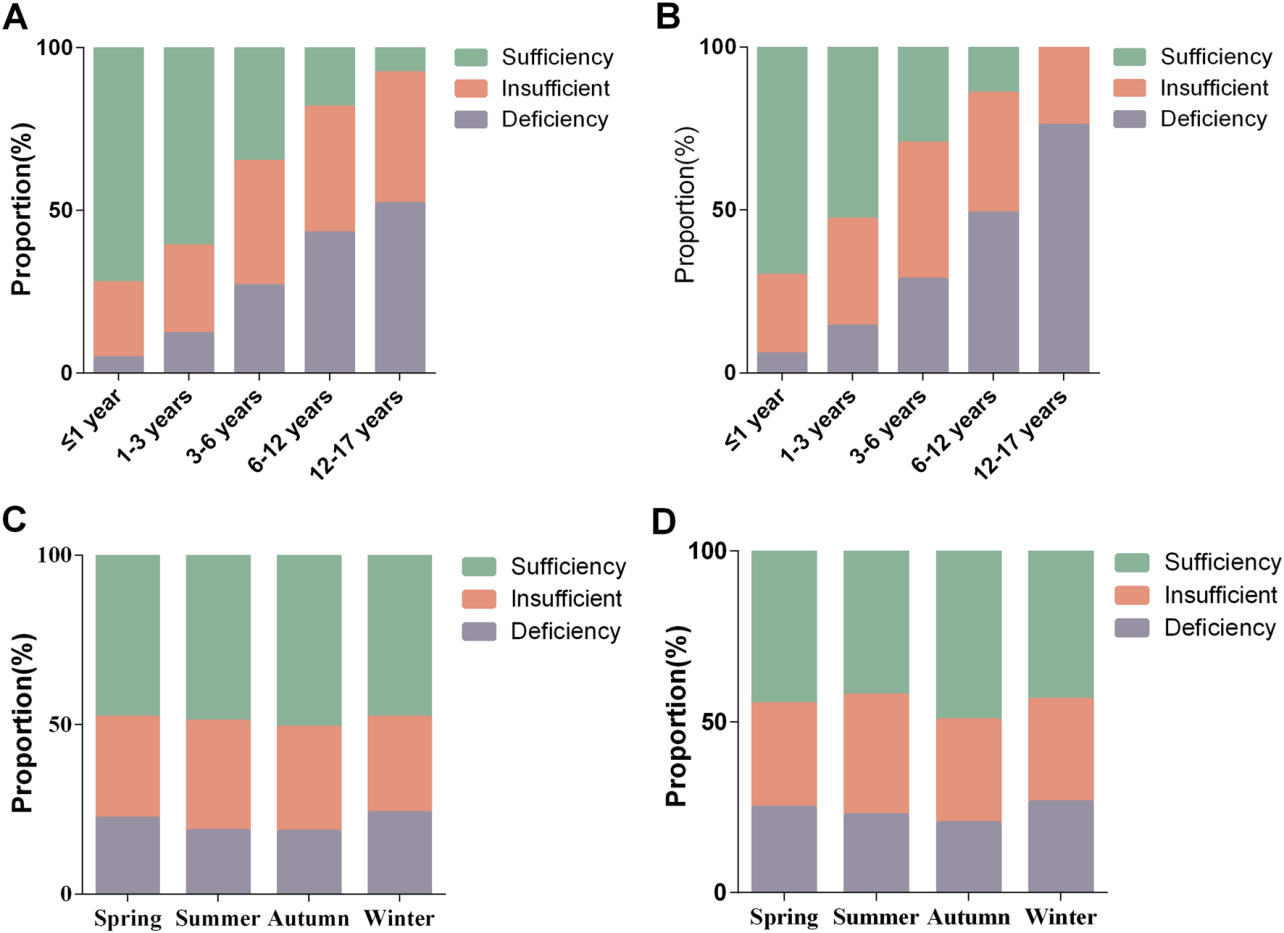
The distribution of serum 25(OH)D levels in boys (**A**) and girls (**B**) in different age groups. The distribution of serum 25(OH)D levels in boys (**C**) and girls (**D**) grouped by different seasons.

In the pediatric population aged 0–17 years, the median serum 25(OH)D concentration was highest during the infancy stage, at 37.36 ng/mL (IQR 28.89–48.00 ng/mL). The serum 25(OH)D concentration decreased with age, with 32.33 ng/mL (IQR 24.70–42.08 ng/mL) in the toddlerhood stage, 25.41 ng/mL (IQR 19.27–32.59 ng/mL) in the preschool-aged group, 20.92 ng/mL (IQR 16.22–27.05 ng/mL) in the school-aged group, and 19.18 ng/mL (IQR 15.31–23.57 ng/mL) in the adolescent group. The serum 25(OH)D concentrations of the children in each age group were significantly different, and those in the preschool age group were already in a state of vitamin D insufficiency (Table 1). Similarly, when analyzing serum 25(OH)D levels across different age groups as stratified by sex, the results indicated that with increasing age, the median serum 25(OH)D levels tended to decrease. Particularly, during the toddler, preschool, school-age, and adolescent stages, boys’ serum 25(OH)D concentrations are significantly greater than those of girls (Fig. 1A). There were significant differences in vitamin D deficiency, insufficiency, and sufficiency among children in each age group (Table 1). The proportions of individuals with a serum 25(OH)D concentration of < 30 ng/mL were 67.3%, 83.4%, and 94.6% in the preschool, school-age, and adolescent periods, respectively. Similarly, in the analysis by sex, as age increased, there was a gradual increase in the number of boys and girls with deficient and insufficient serum 25(OH)D levels (Fig. 2A,B).

### Vitamin D status in different seasonal groups

In the population of children aged 0–17 years, the median serum 25(OH)D concentrations were 29.02 ng/mL (IQR 20.73–39.72 ng/mL) in spring, 28.79 ng/mL (IQR 21.53–39.37 ng/mL) in summer, 30.12 ng/mL (IQR 22.00–39.70 ng/mL) in autumn, and 28.58 ng/mL (IQR 19.97–39.46 ng/mL) in winter. Additionally, there was a statistically significant difference in the serum 25(OH)D concentration in children between autumn and winter, with the median exceeding 30 ng/mL in autumn. The median serum 25(OH)D concentration reached its peak during autumn compared to other seasons. Furthermore, in spring and summer, boys exhibited significantly greater serum 25(OH)D concentrations than girls did (Fig. 1B). There were significant differences in the prevalence of vitamin D deficiency, vitamin D insufficiency, and vitamin D sufficiency in children's serum across the four seasons (Table 1). The proportions of children with sufficient serum 25(OH)D levels across the four seasons were 46.6%, 46.3%, 50.3%, and 46.1%, respectively. Similarly, in the analysis by sex, there was a general trend in the number of boys and girls with deficient and insufficient serum 25(OH)D levels as the seasons changed (Fig. 2C,D).

### Multiple logistic regression analysis model including age, sex, and season

Compared with adolescents aged 12–17 years, children under 1 year old had the lowest risk of vitamin D deficiency/insufficiency in terms of serum 25(OH)D concentration (deficiency: OR 0.005, 95% CI 0.002–0.009; insufficiency: OR 0.046, 95% CI 0.023–0.090) (Table 2). However, as the age increases, the risk of vitamin D deficiency/insufficiency increases. According to the sex-stratified analysis, boys exhibit a lower risk of vitamin D deficiency/insufficiency than girls, with girls serving as the reference group (deficiency: OR 0.729, 95% CI 0.650–0.817; insufficiency: OR 0.837, 95% CI 0.763–0.918) (Table 2). According to the seasonal subgroup analysis, when using winter as the reference, summer results had the lowest risk of vitamin D deficiency/insufficiency (deficiency: OR 0.360, 95% CI 0.304–0.426; insufficiency: OR 0.754, 95% CI 0.656–0.866) (Table 2).

**Table 2:** Association of variables with the 25(OH)D concentration (categorical variable).

Variable		Deficiency				Insufficient				Sufficiency	
		n	%	(95% CI)	P	n	%	(95%CI)	P	n	%
Gender	Boy	1281	53.82%	0.729 (0.650–0.817)	< 0.001	1942	56.22%	0.837 (0.763–0.918)	< 0001	3125	59.16%
	Girl	1099	46.18%	REF		1512	43.78%	REF		2157	40.84%
Age	< 1	222	9.33%	0.005 (0.002–0.009)	< 0.001	1010	29.24%	0.046 (0.023–0.090)	< 0.001	3073	58.18%
	1–3	247	10.38%	0.016 (0.008–0.031)	< 0.001	562	16.27%	0.073 (0.037–0.142)	< 0.001	1106	20.94%
	3–6	532	22.35%	0.064 (0.033–0.125)	< 0.001	768	22.24%	0.177 (0.090–0.347)	< 0.001	633	11.98%
	6–12	1269	53.32%	0.226 (0.117–0.436)	< 0.001	1049	30.37%	0.337 (0.172–0.663)	< 0.001	460	8.71%
	12–17	110	4.62%	REF		65	1.88%	REF		10	0.19%
Season	Spring	546	22.94%	0.735 (0.615–0.878)	< 0.001	705	20.41%	0.924 (0.797–1.073)	> 0.05	1092	20.67%
	Summer	725	30.46%	0.360 (0.304–0.426)	< 0.001	1195	34.60%	0.754 (0.656–0.866)	< 0.001	1656	31.35%
	Autumn	623	26.18%	0.514 (0.434–0.817)	< 0.001	991	28.69%	0.832 (0.725–0.956)	< 0.001	1636	30.97%
	Winter	486	20.42%	REF		563	16.30%	REF		898	17.00%

The multiple logistic regression model includes all the variables in this table.

## Discussion

Vitamin D deficiency and insufficiency are global issues^12^. Our results revealed that the prevalence of serum 25(OH)D deficiency and insufficiency in the 0–17 age group was 21.4% and 31.0%, respectively, emphasizing the prevalence of these issues in children. Furthermore, serum 25(OH)D levels display significant variations across age, sex, and season.

Serum 25(OH)D levels exhibit varying deficiency and insufficiency statuses in different countries and regions and are influenced by varying sunlight exposure. In China, the median concentration of vitamin D generally follows a north–south gradient. Individuals from southern provinces, including Hainan, Guangdong, and Guangxi, exhibit higher levels of 25(OH)D than their counterparts from northern provinces^19^. The median serum 25(OH)D concentration is most elevated in the southernmost province of Hainan and least elevated in the northernmost province of Qinghai^19^. In contrast, in various northern provinces, including Heilongjiang, Jilin, Liaoning, and Inner Mongolia, the 25(OH)D levels surpass those observed in numerous central provinces^19^.

Our study on the impact of age on vitamin D revealed that, in the 0–17 age group of children in Wujiang District, Suzhou City, serum 25(OH)D levels significantly decreased with increasing age. Particularly, in the post-preschool age group, the proportion of individuals with serum 25(OH)D levels < 30 ng/mL exceeded 60%, reaching 90% in adolescence. According to a previous study on vitamin D deficiency in American children, the occurrence of both vitamin D deficiency and insufficiency increases with age, with rates of 14%, 20%, and 28.8% for 2–5-year-olds, 6–11-year-olds, and adolescents, respectively^20,21^. In a study conducted in Harbin, Northeast China, it was found that among children aged 0–12 years, vitamin D levels during early childhood were significantly greater than those during preschool and school-age periods^22^. The prevalence of vitamin D deficiency in children at various stages in Harbin was lower than that in Southeast China^22^. In a Huzhou-based study, 23.28% of children exhibited low vitamin D levels (< 30 ng/mL), with 6.43% experiencing vitamin D deficiency (< 20 ng/mL)^23^. In a Wuxi-based study, the prevalence of vitamin D deficiency (< 20 ng/mL) among preschool children (3–6 years old) was 48.1%, exceeding the rates of 21.2% in the toddler group (1–3 years old) and 17.9% in the infant group (0–1 year old)^24^. When children are older than one year, there is a significant increase in the prevalence of vitamin D insufficiency and deficiency, which corresponds to advancing age^23^. Overall, our study aligns with previous research on vitamin D. However, the adequacy rate of vitamin D levels in children in this region is only 47.5%, with relatively high levels of vitamin D deficiency and insufficiency. Moreover, logistic regression analysis demonstrated that from 1 to 17 years of age, there was a continuous significant decrease in vitamin D deficiency/insufficiency with increasing age. This region is situated at a low latitude, receives abundant sunlight throughout the year, and has a relatively higher economic status than other parts of China. The finding of vitamin D deficiency in this area suggests that parents in the local community may not pay sufficient attention to supplementing their children with vitamin D. Moreover, as children age, there is a gradual increase in the occurrence of vitamin D deficiency and insufficiency, particularly following the school-age period. In light of this observation, we encourage parents to follow the recommendations of the Chinese Nutrition Society, which advocates for a daily supplementation of 400 IU of vitamin D from birth to the age of 50 years^25^.

Additionally, in the infant stage, the adequacy rate of serum 25(OH)D reached 71.4%. Although some infants may experience vitamin D deficiency or insufficiency, vitamin D plays a crucial role in bone development, immune system regulation, cell differentiation, and overall growth during infancy^7^. Maintaining appropriate levels of vitamin D can promote the comprehensive health and development of infants, reducing the risk of certain chronic diseases and infections. Therefore, ensuring sufficient vitamin D intake during infancy is essential for preventing related health issues^1,26,27^. Furthermore, our study revealed that in this region, children exhibit the highest rates of vitamin D deficiency and insufficiency during the winter season. Similar findings have been reported in previous research^28,29^. However, some studies indicate that vitamin D levels gradually increase in the spring and autumn, reaching peak levels in the summer. This trend differs from the findings of our study, in which peak levels were observed in the autumn^22,26^. Although our results indicated a peak in autumn, logistic regression analysis revealed that the risk of vitamin D deficiency/insufficiency was still lowest during summer. Within this region, the lowest prevalence of vitamin D deficiency and insufficiency is observed during the autumn season. This phenomenon may be attributed to the hot summer weather in the region, leading to reduced outdoor activities. Conversely, in the autumn, with a slight decrease in temperature, children engage in more outdoor activities, increasing sunlight exposure, which influences their vitamin D levels. Therefore, we recommend considering supplementation with an appropriate amount of vitamin D, especially during the winter when there is insufficient sunlight and reduced outdoor time.

Regarding sex differences in vitamin D levels, our study revealed a significant difference between boys and girls. Previous research has shown sex differences in vitamin D. However, our results show that the median vitamin D level in boys is 1.24 ng/mL higher than that in girls. Furthermore, logistic regression analysis using girls as the reference indicated that boys have a lower risk of vitamin D deficiency/insufficiency. Other studies have indicated that in the Chinese population, males have higher vitamin D levels than females, ranging from 2.32 to 4.86 ng/mL^30,31^. Likewise, investigations performed in South Korea and the United States have documented lower mean concentrations of 25(OH)D or a greater proportion of vitamin D deficiency among females^32,33^. However, trends reported in Italy and Denmark are exactly the opposite^34,35^. Studies conducted in Germany and Sweden reported no significant differences in vitamin D levels between sexes^13,36^. Likewise, the previously mentioned variations in vitamin D levels between sexes have also been noted in investigations involving Chinese children^22,24,37,38^. Hence, our inclination is to posit that the sex disparities in vitamin D levels are more likely attributed to distinct lifestyles, encompassing dietary patterns and outdoor activity duration, rather than inherent biological differences.

Multiple factors influence children's vitamin D levels, including environmental, behavioral, and physiological factors. Our findings indicate that, in the Chinese population, the prevalence of vitamin D deficiency (< 20 ng/mL) in children aged 0–17 years increases with age. This observation could be attributed to the recommendation of the Chinese Pediatric Society that all children should take in no less than 400 IU/day of vitamin D from birth to 2 years of age^25^. The process of vitamin D conversion in the body includes the synthesis of pre-vitamin D3 through the skin when exposed to ultraviolet light, followed by its conversion to 25-hydroxyvitamin D3 under the action of hepatic dehydrogenase. However, when children are aged 2 and above, the primary source of vitamin D comes from outdoor activities. Research indicates that due to limited outdoor activities, the concentration of vitamin D in children's bodies is lower^38–40^. Based on our research findings and previous studies, as our understanding of children's health continues to grow, we recognize that the situation of vitamin D insufficiency and deficiency is relatively serious in this area. Considering the crucial role of vitamin D levels in the growth and overall health of children, we propose an initiative to supplement vitamin D intake in children. This initiative is intended to ensure that children receive adequate vitamin D support for their well-being.

Our study has notable strengths, including the collection of serum 25(OH)D from 11,116 children aged 0–17 years in Wujiang district, Suzhou city, southeastern China, from 2020 to 2023, providing a substantial sample size. Additionally, we assessed vitamin D levels across different age groups (0–17 years), providing valuable insights into the serum 25(OH)D status based on various factors, such as sex, age, season, and province in this region. However, it is important to acknowledge some limitations in our research. We did not extensively capture the sociodemographic characteristics or lifestyle factors of the children, such as vitamin D supplementation, time spent engaging in outdoor activities, or BMI, which could influence serum 25(OH)D concentrations. Nevertheless, the study population comprised 11,116 children undergoing routine health check-ups at hospitals, enhancing the representativeness of participants within the local child population. Further research is warranted to explore potential factors and variables associated with vitamin D status^41^.

## Methods

### Ethical approval

Informed consent was obtained from the parents/legal guardians before their children participated in the study, and assent was obtained from all children aged 7 years or older. The research protocol was developed in accordance with the ethical guidelines of the Ethics Review Committee of Children's Hospital of Wujiang District, Suzhou City (NO:2023010).

### Participants

Suzhou, Wujiang district is located within the administrative jurisdiction of Suzhou city in Jiangsu Province, with geographical coordinates of approximately 31.1355 degrees north latitude and 120.6469 degrees east longitude. The region has a subtropical monsoon climate characterized by hot and humid summers and cold and damp winters, with moderate temperatures throughout the year. Sunshine duration varies due to seasonal changes, but on average, Wujiang district in Suzhou experiences approximately 4–6 h of sunshine per day throughout the year.

From January 2020 to November 2023, we collected data from all children undergoing routine vitamin D testing during outpatient visits throughout the year. The data were organized by season: spring (March to May), summer (June to August), autumn (September to November), and winter (December to February). Comprehensive clinical information, including patient name, age, sex, and blood collection date, were gathered for all participants. Based on the children's ages, they were categorized into five groups: infancy (0–1 year), toddlerhood (1–3 years), preschool age (3–6 years), school age (6–12 years), and adolescence (12–17 years). In alignment with serum 25(OH)D levels, the examined children were classified into three groups: 25(OH)D deficiency (25(OH)D < 20 ng/mL), insufficiency (25(OH)D = 20–30 ng/mL), and sufficiency (25(OH)D > 30 ng/mL)^42^. This classification provides a comprehensive understanding of vitamin D status among the children in different age groups and across seasons.

### Vitamin D assay methods

Vitamin D testing is a routine diagnostic procedure conducted in outpatient settings for children utilizing liquid chromatography‒mass spectrometry/mass spectrometry (LC–MS/MS) technology. The ACQUITY UPLC/TQS instrument (Waters, USA) was used. Initially, 2 mL of peripheral blood was collected from the patient and centrifuged to obtain high-purity serum samples. Subsequent sample processing involves washing steps to remove potential interferences. Liquid chromatography separation techniques are then applied, in which the sample traverses a chromatographic column for separation, eliminating interfering substances. Ultimately, mass spectrometric analysis is performed with a mass spectrometer to ascertain the concentration of 25(OH)D in the serum through the interpretation of the mass spectrum. To ensure accuracy, data analysis is typically performed in conjunction with a standard curve^43^.

### Statistical analysis

The distributions of the children's serum 25(OH)D levels were skewed; hence, the median and interquartile range (IQR) were used to represent the concentration of vitamin D. Chi-square tests were used to compare the differences in vitamin D levels among different populations based on categorical variables, considering the frequency and percentage of the classified variables. The Mann–Whitney U rank-sum test was used for median comparisons between two independent samples, while the Kruskal–Wallis H test was applied for median comparisons among multiple independent samples and pairwise comparisons. A multinomial logistic regression analysis was performed on the variables representing vitamin D status. All the statistical analyses had a significance threshold of *P* < 0.05. The analyses were conducted using SPSS (version 26.0), and data visualization was performed using GraphPad Prism software.

### Institutional review board statement

The study was conducted in accordance with the Declaration of Helsinki, and approved by the Ethics Committee of Children's Hospital of Wujiang District, Suzhou City (NO:2023010) for studies involving humans.

### Informed consent

Informed consent was obtained from all subjects involved in the study.

## Data Availability

Data can be provided by the corresponding author upon reasonable request.
